# Athlete or Non-athlete? This Is the Question in Body Composition

**DOI:** 10.3389/fphys.2021.814572

**Published:** 2021-12-17

**Authors:** Francesco Campa, Giuseppe Coratella

**Affiliations:** ^1^Department for Life Quality Studies, Università degli Studi di Bologna, Bologna, Italy; ^2^Department of Biomedical Sciences for Health, Università degli Studi di Milano, Milan, Italy

**Keywords:** BIA, BIVA, resistance training, phase angle, predictive equations

## Introduction

The term “athlete” is used worldwide to indicate a given population, albeit it is not clear where and when it originated. This word comes from the Greek root “Athlos” which means “achievement” or “contest” and a more complex figure than just the simple sportsman, since he/she embodied the greatest virtues of a human being. The first testimony of the term athlete is found in the Odyssey, when Ulysses was mocked by Feaci for not wanting to participate in sports competitions, and they accused him of being greedy, lacking virtue and consequently not being an athlete (Homer, 1997).

To date, several organizations including the American Heart Association and the European Society of Cardiology offer definitions that emphasize “organized competition” and an “award for excellence and success” as integral components in the definition of an athlete (Maron and Zipes, 2005; Pelliccia et al., 2005). The American Heart Association defines an athlete as “one who participates in organized team or individual sports that require regular competition against others as a core component and places a high value on excellence and achievement, requiring some form of systematic training (usually intense)” (Maron and Zipes, 2005). Similarly, the European Society of Cardiology defines an athlete as “an individual of young or adult age, amateur or professional, who is engaged in regular physical training and participates in official competitions” (Pelliccia et al., 2005). Recently, Araújo and Scharhag (2016) proposed that the intent of the physical activity is the primary feature that distinguishes an exerciser from an athlete, identifying four criteria that should be simultaneously fulfilled to define a person as an athlete: (i) to be training in sports aiming to improve his/her performance or results; (ii) to be actively participating in sport competitions; (iii) to be formally registered in a local, regional or national sport federation as a competitor; and (iv) to have sport training and competition as his/her major activity or focus of interest, almost always devoting several hours a day to these sport activities, exceeding the time allocated to other professional or leisure activities. These points were later shared and updated, defining athletes as “people who engage in physical activity with the primary goal of improving performance to bolster athletic excellence and/ or achievement” (MacMahon and Parrington, 2017).

On the other hand, exercisers should be identified as people who participate in physical activity with the motivation to increase fitness, promote health, improve physique, and learn or refine skills. Subsequently, McKinney et al. (2019) in an editorial article titled “Defining athletes and exercisers” supported how the intent of the training should be a key criterion for discerning an athlete from an exerciser. In addition, the authors suggest considering the “volume of exercise” (hours/week) as a quantitative metric that further allows the stratification of athletes and the “level of competition” as a further criterion to help to define groups of people who fulfill the criteria of an athlete (McKinney et al., 2019). Accordingly, “elite” athletes are defined as individuals who exercise >10 h/week and whose athletic performance has achieved the highest level of competition, “competitive” athletes exercise >6 h/week with an emphasis on improving performance, “recreational” athletes exercise >4 h/week for unregulated competitions, while an exerciser engages in >2.5 h/week of physical activity with the primary aim to maintain health and fitness status (McKinney et al., 2019).

However, with the aim of distinguishing athletes from exercisers, none of the current classifications has considered the body composition characteristics. Intriguingly, an individual who participates in a marathon with the purpose of running for leisure and therefore classified as an exerciser may show the same body composition features of a subject who runs the same race with the intent of competing and therefore considered an athlete. Similarly, a person who carries out manual works such as farmers, builders, or a gym attender that does not compete in organized events or is not affiliated with a sports team could nevertheless have similar body composition characteristics to those of an athlete. Such a lack of clarity could cause confusion when attempting to distinguish athletes from exercisers and may have both scientific and practical repercussions. In the present opinion paper, we will discuss why the current definition of athlete can lead to an incorrect assessment of body composition, providing suggestions and future perspectives to fill this gap.

### Why Is the Current Definition of “Athlete” a Problem in Body Composition Assessment?

The assessment of body composition is crucial in different contexts. In research, it is used to evaluate the benefits of a training strategy or the effects of aging or growth, as well as the effectiveness of nutritional strategies (Fornetti et al., 1999; Matias et al., 2016, 2021; Sardinha et al., 2020; Campa et al., 2021; Kasper et al., 2021; Lukaski and Raymond-Pope, 2021). In practice, nutritionists, medical doctors, or trainers evaluate body composition to set nutritional intervention strategies or training programs. The body composition components can be accurately examined through densitometric (hydrostatic weighing and displacement plethysmography), imagine (dual-energy X-ray absorptiometry, magnetic resonance, and computed tomography) or dilution techniques (Campa et al., 2021). However, these methods are expensive, non-transportable and require long measurement times in addition to specialized personnel. For this reason, low-cost and user-friendly techniques are often preferred in both sports research and practice.

In these contexts, the bioelectrical impedance analysis is widely used to quantify body composition elements (e.g., fat and fat-free mass, body fluids, muscle mass) and are based on predictive equations developed comparing with densitometry, imaging and dilution techniques as reference (Campa et al., 2021; Lukaski and Raymond-Pope, 2021). Starting from the unique impedance properties of each tissue, several regression equations have been implemented to obtain several body composition parameters. Particularly, the impedance includes the resistance, the force that a biological conductor opposes to an alternating current attributable to intracellular and extracellular fluids, and the reactance, arising from the cell membranes and representing the capacitive component of the impedance (Campa et al., 2021). Depending on the resistance and reactance of each tissue, different body composition components can be determined. To date, several predictive equations have been developed considering different populations (Campa et al., 2021; Coratella et al., 2021), and importantly different predictive equations provide different outcomes when used on the same subjects (Pichard et al., 1997; Coratella et al., 2021). Therefore, an accurate choice of predictive formulas is needed.

The current definition of an athlete may represent a problem when using bioelectrical impedance analysis for assessing body composition. Despite their body composition features, some exercisers could not have the requisites to be defined as athletes, and consequently body composition might be estimated less accurately using equations developed for the general population. In this regard, the error would derive from the fact that a group of exercisers, having similar body composition characteristics to those of the subjects involved in the studies that have validated the formulas for athletes, would result in greater accuracy when equations for athletes are used (Coratella et al., 2021). Unfortunately, body composition characteristics cannot be discovered until a method of analysis is applied. More specifically, unless one relies on a subjective evaluation, there are no pre-screening indices that have been proposed to help practitioners to overcome this problem. Consequently, practitioners simply use equations for athletes when specific so-defined athletes are tested or generalized equations when exercisers or different populations are involved. Furthermore, according to the instructions provided by some manufacturers of the bioelectrical impedance analyzer devices, the equations for athletes should be chosen when facing with a subject with a “heart rate below 60, or training 3 times a week.” However, comparative studies showed that these criteria may not represent a valid cut-off (Loenneke et al., 2013). As such, it appears necessary to propose simple procedures to help both scientists and practitioners to choose specific formulas for athletes, even exercisers are tested.

### What We Propose

When using bioimpedance analysis, the phase angle is a parameter that faithfully reflects the ratio between intra and extracellular fluids, as well as the cell integrity, and is derived from the relation between the direct measure of bioelectrical resistance and reactance (Campa et al., 2020c). Particularly, the electric current passing through the body will flow through two different pathways: the extracellular pathway and the intracellular pathway (Lukaski and Piccoli, 2012). In the extracellular pathway, the current will be conducted through the interstitial fluid and plasma, which will offer a resistance inversely proportional to the fluid and electrolyte content (Lukaski and Piccoli, 2012). In the intracellular pathway, the intact cell membranes will act as a capacitive element, storing some energy and delaying the current passage, which then becomes out-of-phase (Lukaski and Piccoli, 2012). This delay, or phase shift, is expressed as phase angle, measured directly by a phase-sensitive bioelectrical devices (Lukaski and Raymond-Pope, 2021). Then phase angle has the advantage of being directly estimated from the raw bioelectrical measurements, without the need for weight, height, or any other conversion equation. Previous studies have shown that phase angle is higher in athletes than in the general population and that its value is positively correlated with muscle mass and the intracellular/extracellular water ratio (Campa et al., 2021; Lukaski and Raymond-Pope, 2021). Since athletes are expected to show greater muscle mass and intracellular/extracellular water ratio, phase angle could be initially assessed as a pre-screening index. Exercisers and athletes with similar body composition could show a similar phase angle, we suggest setting specific thresholds to define when generalized or formula for athletes should be used. With this in mind, we and other authors also have encouraged researchers and practitioners to personally choose the predictive equation where the raw bioelectrical parameters should be inserted, instead of using the software provided by the manufacturers (Campa et al., 2021; Lukaski and Raymond-Pope, 2021).

Another option might be using the bioelectrical impedance vector analysis (BIVA) to compare the position of the vector with respect to the references of the general and athletic population. By doing so, it could be possible to identify when an exerciser ranks closer to the average values of athletic or generalized population using the population-specific BIVA ellipses and their percentiles as reference. Particularly, BIVA does not provide estimates of volume or mass, but a classification (e.g., more or less body fluids or fat mass) and ranking (e.g., better or worse after treatment or intervention) tool (Lukaski and Raymond-Pope, 2021). In this regard, previous studies have already shown how groups of exercisers can present BIVA patterns similar to those of athletes (Campa et al., 2020a,b). Therefore, evaluating the position of the BIVA vector may be an initials screening to choose the most accurate predictive equation.

Lastly, given that the characteristics of body composition deeply vary depending on the sport (Santos et al., 2014; Campa et al., 2019a,b), specific formulas for each type of sport should be developed. However, we acknowledge that the development and the validation of specific equations for each sport represents a great challenge for researchers, who must select large sample sizes and considering sex, age, ethnicity, level, and competitive period as independent factors.

## Conclusions

**Figure 1:**
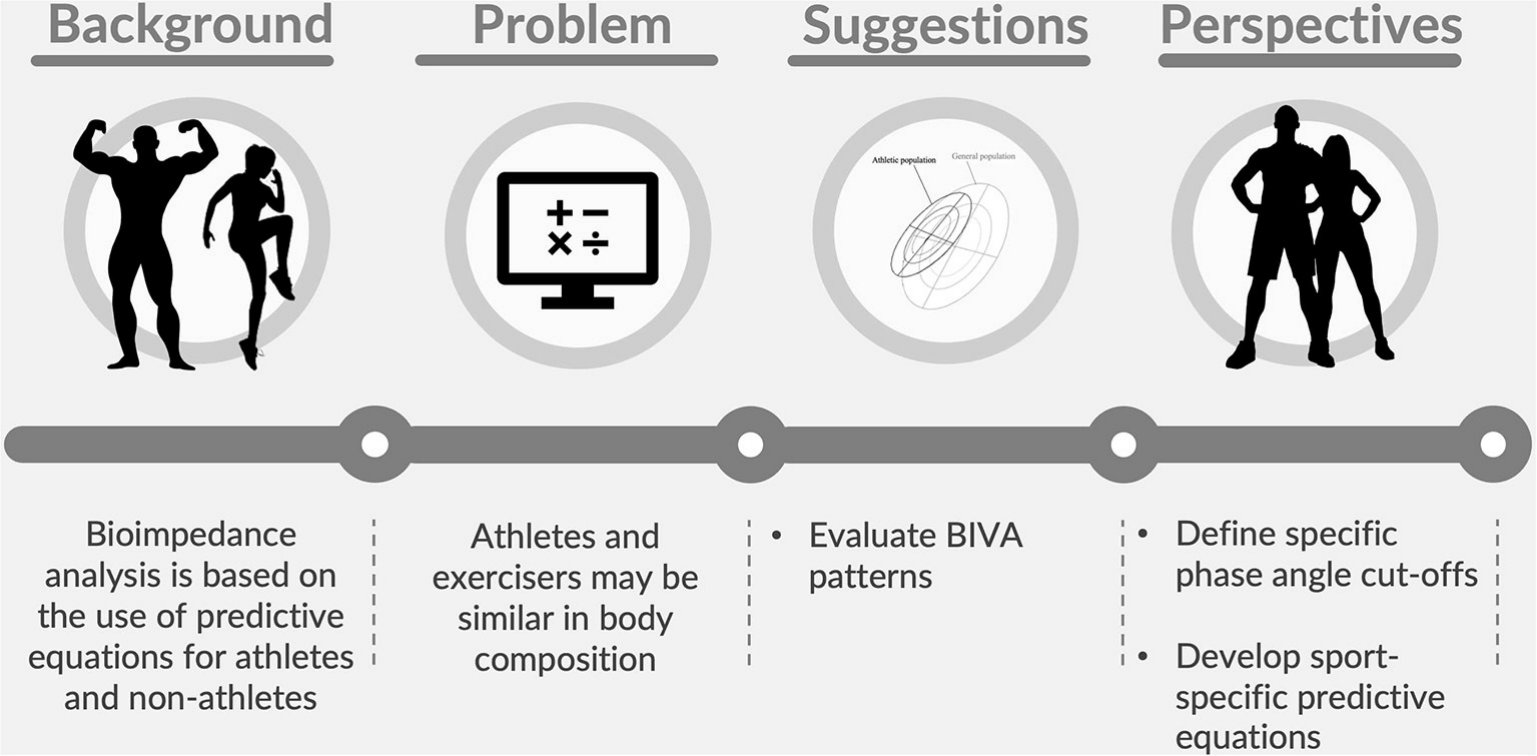
The schematical representation of the problem, proposed solutions, and future perspectives related to the body composition assessment in exercisers.

The current definitions of athlete identify people engaged in competitive sporting events individually or in teams, with high physical performance and specific training methods (Araújo and Scharhag, 2016; McKinney et al., 2019). However, this excludes a wide range of individuals who train recreationally but may still present body composition characteristics similar to those of an athlete. When body composition is evaluated using bioimpedance analysis, we have proposed some parameters that should assessed with the intent of using the most accurate predictive equations. Purposively, the evaluation of the phase angle and the vector position through BIVA could represent a solution to the problem, thus determining whether a person appears closer to the body composition characteristics of the athletic rather than the general population. Figure 1 schematically summarizes the problem, proposed solutions, and future perspectives. The current work calls for action researchers to propose adequate methods for distinguishing athletes from exercisers, a key-point when body composition is assessed using bioimpedance analysis.

## Author Contributions

FC and GC: conceptualization, writing-original draft, and writing-review and editing. All authors contributed to the article and approved the submitted version.

## Conflict of Interest

The authors declare that the research was conducted in the absence of any commercial or financial relationships that could be construed as a potential conflict of interest.

## Publisher's Note

All claims expressed in this article are solely those of the authors and do not necessarily represent those of their affiliated organizations, or those of the publisher, the editors and the reviewers. Any product that may be evaluated in this article, or claim that may be made by its manufacturer, is not guaranteed or endorsed by the publisher.
